# Hypoxia attenuates Hsp90 inhibitor 17-DMAG-induced cyclin B1 accumulation in hepatocellular carcinoma cells

**DOI:** 10.1007/s12192-015-0664-2

**Published:** 2016-01-20

**Authors:** Jianming Zhang, Huadan Li, Zhizhou Huang, Yangfan He, Xueqiong Zhou, Tingyuan Huang, Peijuan Dai, Danping Duan, Xiaojiao Ma, Qiangbin Yin, Xiaojie Wang, Hong Liu, Size Chen, Fei Zou, Xuemei Chen

**Affiliations:** Department of Occupational Health and Medicine, School of Public Health and Tropical Medicine, Southern Medical University, 1838 Guangzhou Road North, Guangzhou, 510515 China; Department of Oncology, The First Affiliated Hospital of Guangdong Pharmaceutical University, 19 Nonglinxia Road, Guangzhou, 510080 China

**Keywords:** Hypoxia, 17-DMAG, Chemoresistance, Cyclin B1, Cell cycle arrest

## Abstract

Hypoxia stress plays a pivotal role in tumor formation, proliferation, and invasion. Conventional chemotherapy is less effective in the hypoxia microenvironment of solid tumor. Heat shock protein 90 (Hsp90) is an important molecular chaperone in cancer cells and has been a pharmaceutical target for decades. However, Hsp90 inhibitors demonstrate limited effect on solid tumor and the mechanism underlying is not clear. To determine whether hypoxia impairs the therapeutic effect of Hsp90 N-terminal inhibitor, 17-demethoxygeldanamycin hydrochloride (17-DMAG), in live cancer cells, we measured cell proliferation and cell cycle distribution. Cell proliferation assay indicates that hypoxia obviously promotes the proliferation of HepG2 and Huh7 cells after 24, 48, and 72 h and impairs 17-DMAG-induced G2/M arrest in liver cancer cells. As a client protein of Hsp90, cyclin B1 is critical for the transition from G2 to M phase and is related to the prognosis of the patients. We further checked the cyclin B1 messenger RNA (mRNA) level, protein level, ubiquitination of cyclin B1, nuclear translocation, and degradation of cyclin B1 affected by hypoxia after 17-DMAG treatment. The results demonstrate that hypoxia decreases the transcription of cyclin B1 and accelerates the ubiquitination, nuclear translocation, and degradation of cyclin B1. Taken together, our results suggest that hypoxia attenuates cyclin B1 accumulation induced by 17-DMAG and, hence, alleviates 17-DMAG-induced G2/M arrest.

## Introduction

Hepatocellular carcinoma (HCC) is one of the most common malignancies and the second most frequent cause of cancer mortality worldwide (Lin et al. 2012). According to 2014 Cancer Country Profiles from World Health Organization, 394,770 of new liver cancer cases and 380,772 cancer deaths occurred in China. Besides China, the number of cases diagnosed with HCC is expected to increase in western countries (Venook et al. 2010). In China, 5-year relative survival rate of liver cancer is only 10.1 % (Zeng et al. 2015).

Identification of novel therapeutic agents via molecular targeting offers the promise of treatment for advanced liver cancer. However, the therapy targeted for one single molecular faces the potential peril of being subverted by the inherent genetic plasticity of cancer cells. Multiple molecular targets have been used in combination, which are involved in a multitude of signaling pathways leading to simultaneous adverse effects on oncogenic proteins, would prove promising, but also would make therapy more complicated and prolong the trials (Patki and Pawar 2013; Isaacs et al. 2003).

Heat shock protein 90 (Hsp90), as an important member of the heat shock protein family, is usually constitutively expressed in most mammalian cell types and can be further induced by heat shock and other stresses. Hsp90 plays a vital role in assisting more than 200 client proteins for their proper folding, stability, and function (Patki and Pawar 2013). The protein has been championed for over 20 years by the National Cancer Institute (Bethesda, MD, USA) as a cancer target since the discovery of the antitumor activity of the natural product geldanamycin (Barrott and Haystead 2013). So far, many kinds of Hsp90 inhibitors have been identified and many of them have already exhibited good antitumor effects and have entered into different stage of clinical trials, such as geldanamycin (GA), 17-(allyamino)-17-demethoxygeldanamycin (17-AAG),17-dimethylaminoethylamino-17-demethoxygeldanamycin hydrochloride (17-DMAG), and so on. The crystal structure reveals that 17-DMAG binds to the ATP binding site of Hsp90 N-terminal domain and is currently in phase I clinical trials (Pacey et al. 2011). However, the therapeutic effects vary in tumors, especially in solid tumor including liver cancer, which presents poor response to Hsp90 inhibitors.

Since the hypoxia microenvironment of the solid tumor cells impairs the sensitivity to traditional chemotherapy, new chemotherapies of liver cancer treatment are being sought. Hypoxia is a characteristic of diverse human solid tumors, including liver cancer, breast cancer, prostate cancer, and pancreatic cancer (Harrison and Blackwell 2004). especially, in the processes of tumor growth. A rapidly growing tumor mass quickly outstrips its vasculature and lacks oxygen and nutrients. Although hypoxia is a harmful factor to the most of normal cells and some tumor cells, it also provides a strong selective pressure for the survival of the most aggressive and metastatic cells (Yan et al. 2009). In fact, clinical studies have clearly shown that there are two sides of the hypoxia. On one hand, hypoxia induces unfolded protein response, cell death, and the DNA damage response (DDR) (Olcina et al. 2010). On the other hand, hypoxia is a plus factor to HCC. This situation is therapeutically significant, as hypoxic cells are more resistant to both chemotherapy and radiotherapy (Black et al. 2015; Karakashev and Reginato 2015; Horsman et al. 2012; Ghattass et al. 2013). In addition, hypoxia promotes HCC invasion, metastasis (Yan et al. 2012). and proliferation (Gwak et al. 2005).

Based on these previous studies of chemotherapy under normoxia and hypoxia, we raised the hypothesis that hypoxia could decrease the chemoresistance of HCC cells to 17-DMAG. To test our hypothesis, we examined the effects of normoxia or hypoxia on cell viability after 17-DMAG treatment. We found that hypoxia counteracted 17-DMAG-induced cell proliferation inhibition and attenuated the mitotic blockage in G2/M with decreased cyclin B1.

Cyclin B1 is a client protein of Hsp90 and also a key protein in the control of cell cycle transition from G2 to M phase (Ortiz et al. 2002). Deregulated expression of cyclin B1 may induce a disrupted control of cell growth and a malignant phenotype (Egloff et al. 2006; Chen et al. 2009). Hence, the mechanism of the level change of cyclin B1 affected by hypoxia and 17-DMAG treatment in HCC cells was further explored in this paper.

## Materials and methods

### Cell culture and reagents

Human liver cancer cell line HepG2 and Huh7 cells were cultured in Dulbecco’s modified Eagle’s medium (DMEM) medium with 10 % fetal bovine serum (FBS), 100 units/ml penicillin, and 100 μg/ml streptomycin. Normoxia or hypoxia conditions were maintained at 37 °C in the incubator with 20 % O_2_ and 5 % CO_2_ or 95 % N_2_, 5 % CO_2_, and 1 % O_2_. Hsp90 inhibitor 17-DMAG (Selleck, Houston, TX, USA), anti-Hsp90α SPA840 (Enzo, Farmingdale, NY, USA), a mouse anti-monoubiquitinylated and polyubiquitinylated conjugate antibody, clone FK2 (Merck Millipore, USA), a rabbit anti-ubiquitin antibody Ub antibody (FL-76) (Santa Cruz, USA), a rabbit anti-cyclin B1 antibody (H-20) (Santa Cruz, USA), and the Cell Cycle Regulation Antibody Sampler Kit II (CST, Danvers, MT, USA) were purchased. IRDye secondary antibodies (Li-Cor, Lincoln, NE, USA) were used in the experiments.

### Cell viability assay

Cell viability was measured with a cell counting kit-8 (CCK8) assay (Dojindo, Tabaru, Mashikimachi, Japan). Briefly, HepG2 and Huh7 cells were seeded at a density of 2 × 10^3^ cells per well in 96-well plates and incubated for 24 h. The cells were treated with different concentrations of 17-DMAG (0, 0.01, 0.05, 0.1, 0.5, 1, 5, 10 μM) in normoxia or hypoxia. After the cells were incubated for 24 or 48 h, the CCK-8 solution was added to each well for another 2 h at 37 °C. The absorbance was measured with a 96-well plate reader at 450 nm. Six replicate wells were included in each group.

### Cell cycle analysis

Huh7 and HepG2 cells were incubated with 17-DMAG (0, 0.05, 0.1, 0.5, 1 μM) for 24 h under normoxia or hypoxia condition. Cells were harvested and washed once in phosphate-buffered saline (PBS). The resuspended cells were added dropwise into a 15-ml conical tube containing 5 ml of ice-cold 70 % ethanol while vortexing on medium speed. Cells were kept at −20 °C overnight. Samples then were washed with PBS and incubated with RNase and propidium iodide solution for 30 min at 37 °C. The DNA content was analyzed by GUAVA flow cytometry (Millipore, Billerica, MA, USA). The percentage of cells in each phase of the cell cycle was determined by Mod fit LT 4.0 (Verity Software House, Topsham, ME, USA).

### Western blot analysis and co-IP

The cell lysis buffer consisted of 10 mM Na_2_HPO_4_, 1.8 mM KH_2_PO_4_ pH 7.4, 137 mM NaCl, 2.7 mM KCl, 1 % Nonidet P-40, 0.5 % deoxycholate, 0.3 % sodium dodecyl sulfate (SDS), 1 mM sodium orthovanadate, and 1 mM phenylmethylsulfonyl fluoride. Protein concentrations of the cell lysates were determined by the BCA method (Bio-Rad). Each sample (40 μg) was separated by 10 % SDS-PAGE and transferred onto a PVDF membrane. The membrane was blocked in Tris-buffered saline (TBS) plus 5 % BSA for 90 min at room temperature and then incubated with primary antibody in TBST plus 1 % BSA overnight at 4 °C. The membrane was washed four times with TBST (TBS containing 0.1 % Tween 20) for 5 min each time at room temperature and then incubated with IRDye 680/800 secondary antibody for 60 min. After washing the membrane six times with TBST for 5 min each time at room temperature, immunoreactive bands were detected by Odyssey Infrared Imaging System (Li-Cor, Lincoln, NE, USA).

For co-immunoprecipitation (co-IP), the proteins with primary antibody, a mouse anti-monoubiquitinylated and polyubiquitinylated conjugate antibody, and clone FK2 (Merck Millipore, USA) were incubated in scroll oscillator overnight at 4 °C; then, protein G agarose beads were added to the mixtures and incubated for another 2 h. The agarose beads were washed three times with cell lysis buffer and then boiled with 2× SDS protein sample loading buffer for 10 min. A rabbit anti-ubiquitin antibody Ub antibody (FL-76) (Santa Cruz, USA) and rabbit anti-cyclin B1 antibody (H20) (Santa Cruz, USA) were used for Western blot to avoid the inference of mouse IgG heavy chain and light chain in this study.

### Quantitative real-time reverse transcription polymerase chain reaction assay

HepG2 and Huh7 cells were harvested in 35-mm dishes, and RNA was isolated by TRIzol (Takara, Otsu, Shiga, Japan). Five hundred nanograms of RNA was applied to synthesis first-strand complementary DNA and using first-strand cDNA Synthesis Kit (Takara, Otsu, Shiga, Japan). Reverse transcription polymerase chain reaction (RT-PCR) was done following the manufacturer’s instructions by Mx3000P Real-Time PCR Detection System (Stratagene, Santa Clara, CA, USA) with a mixture composed of SYBR Green (Takara, Otsu, Shiga, Japan), 1 μl (0.2 μmol/l) of each primer, and 1.6 μl of complementary DNA from RT-PCR samples. The primer sequences were as follows: β-actin, 5′-TGGCACCACACCTTCTACAAT-3′ (forward), 5′-AGAGGCGTACAGGGATAGCA-3′ (reverse); cyclin B1, 5′-ACCAAAATACCTACTGGGTCGG-3′ (forward), 5′-GCATGAACCGATCAATAATGG-3′ (reverse). The level of cyclin B1 messenger RNA (mRNA) was quantified by the Ct values, and β-actin was set as an internal control. Experiments were performed in triplicate. The results were analyzed by the ∆∆CT method.

### Stable transfection of mCherry-cyclin B1 and time-lapse imaging

With 20 μg plasmid DNA for the expression of cyclin B1-mCherry wild-type (WT) (Addgene, Cambridge, MA, USA) or empty control vector at 306 V, 975 μF, and 25.8 ms in 0.4-cm electroporation cuvettes (Bio-Rad, Hercules, CA, USA) by Gene Pulser II Electroporation System (Bio-Rad, Hercules, CA, USA), 1 × 10^7^ HepG2 cells were electroporated. Selection was initiated 48 h after transfection using 1000 μg/ml of G418 (Life Technologies, Grand Island, NY, USA). The selection medium was changed every 2 days for 4 weeks until all control cells died. Resistant single-cell clones were isolated and digested under inverted fluorescent microscopy and cultured for further identification.

HepG2 cells stably transfected with mCherry-cyclin B1 were seeded in 35-mm glass bottom dishes. After serum starvation for 24 h to achieve cell synchronization, cells were kept at 0.5 μM 17-DMAG in DMEM medium with 10 % FBS at 37 °C in cell chamber supplied with mixed gas supply to mimic normoxia condition (5 % CO_2_, 95 % air) or hypoxia condition (1 % O_2_, 5 % CO_2_, 94 % N_2_), respectively. Time-lapse images were obtained by Olympus FV1000 confocal microscope (×40) (Olympus, Tokyo, Japan). The mCherry-cyclin B1 images were obtained every 15 min for 24 h. Quantifications for total and nuclear cyclin B1 were performed using ImageJ software 1.48 (National Institutes of Health, Bethesda, MD, USA). For cyclin B1 intensity measurements, we used the following formulas: whole-cell signal = sum of the intensity of the pixels for one cell; nucleus signal = sum of the intensity of the pixels for nucleus portion; cytoplasm signal = sum of the intensity of the pixels for one cell − sum of the intensity of the pixels for nucleus; background signal = mean signal per pixel for a region selected just beside the cell; whole-cell signal corrected = whole-cell signal − (area for the selected cell × mean background); nucleus signal and cytoplasm signal were also corrected (Gavet and Pines 2010).

### Statistical analysis

Results are presented as the mean ± standard error of the mean (SEM). Statistical analyses were performed using the Student’s *t* test or one-way analysis of variance test with SPSS 13.0 (IBM, Armonk, NY, USA). Graphs were generated with Excel. *P* < 0.05 was denoted as statistically significant.

## Results

### Hypoxia promoted proliferation and chemoresistance to Hsp90 inhibitor 17-DMAG in HCC cells

**Fig. 1:**
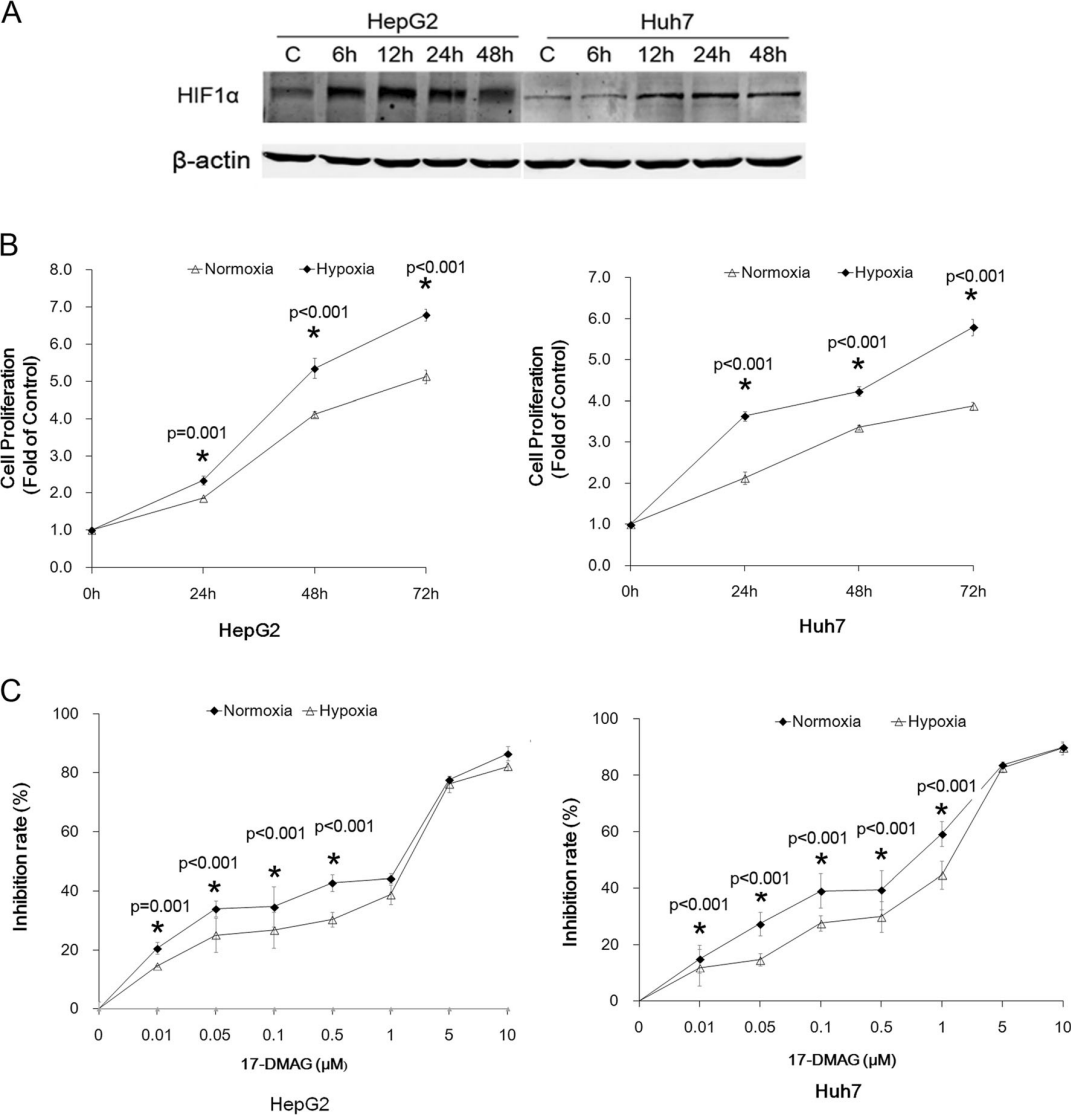
Hypoxia promoted proliferation and chemoresistance to Hsp90 inhibitor 17-DMAG in hepatocellular carcinoma cells. HepG2 and Huh7 cells were grown under normoxia (20 % O_2_ and 5 % CO_2_, 37 °C) or hypoxia (1 % O_2_, 5 % CO_2_, and 94 % N_2_, 37 °C). CCK-8 assay was performed according to the manufacturer’s instruction. **a** Hypoxia effect was confirmed by the increased level of HSF1. **b** Hypoxia promoted cell proliferation both in HepG2 and in Huh7 cells. **c** The proliferative inhibition effect of 17-DMAG on HepG2 and Huh7 cells under normoxia and hypoxia after 24-h treatment with 17-DMAG. The inhibition rate was significantly decreased under hypoxia. Data are presented as mean ± SD of three independent experiments (*p < 0.05, hypoxia vs. normoxia)

HepG2 and Huh7 cells were cultured under normoxia or hypoxia condition. The effect of hypoxia treatment was examined by HIF1α level (Fig. 1a). Cell proliferation assay indicated that hypoxia obviously promoted the proliferation of HepG2 and Huh7 cells after 24, 48, and 72 h (Fig. 1b). We then treated HepG2 and Huh7 cells with different concentrations of 17-DMAG (0.01, 0.05, 0.1, 0.5, 1.0, 5.0, and 10 μM) for 24 h. As shown in Fig. 1c, Hsp90 inhibitor 17-DMAG significantly inhibited cell proliferation and was displayed in a concentration-dependent manner. However, compared with normoxia, hypoxia caused the decreased susceptibility to 17-DMAG, especially under a concentration of 1.0 μM. The calculated half-maximal growth inhibition concentration (IC50) after 24-h treatment of 17-DMAG was 0.51 vs. 0.31 μM (normoxia vs. hypoxia) in HepG2 cells and was 0.89 vs. 0.76 μM (normoxia vs. hypoxia) in Huh7 cells.

### Hsp90 inhibitor 17-DMAG induced cell cycle arrest at G2/M phase and hypoxia attenuated the arrest

**Fig. 2:**
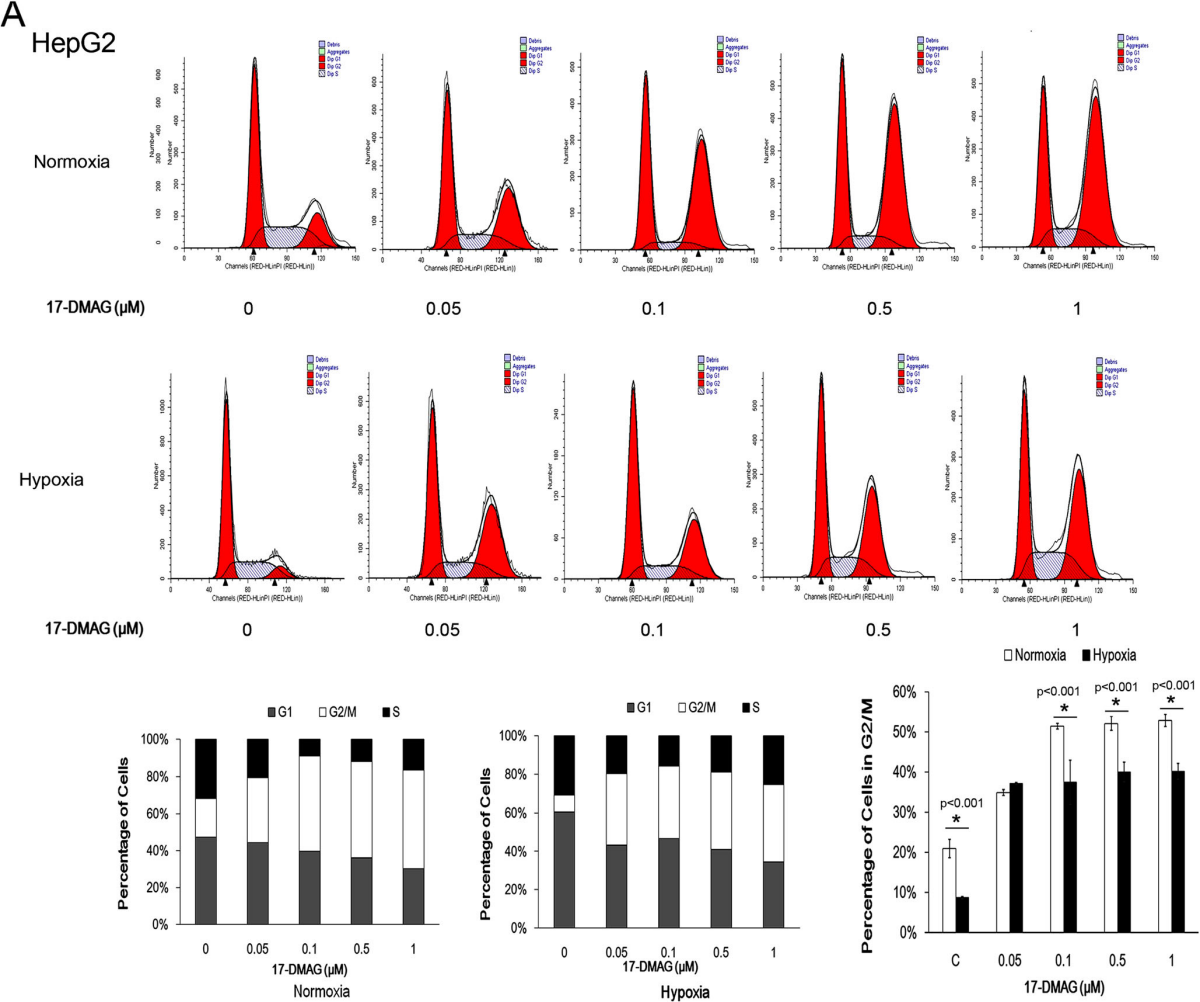

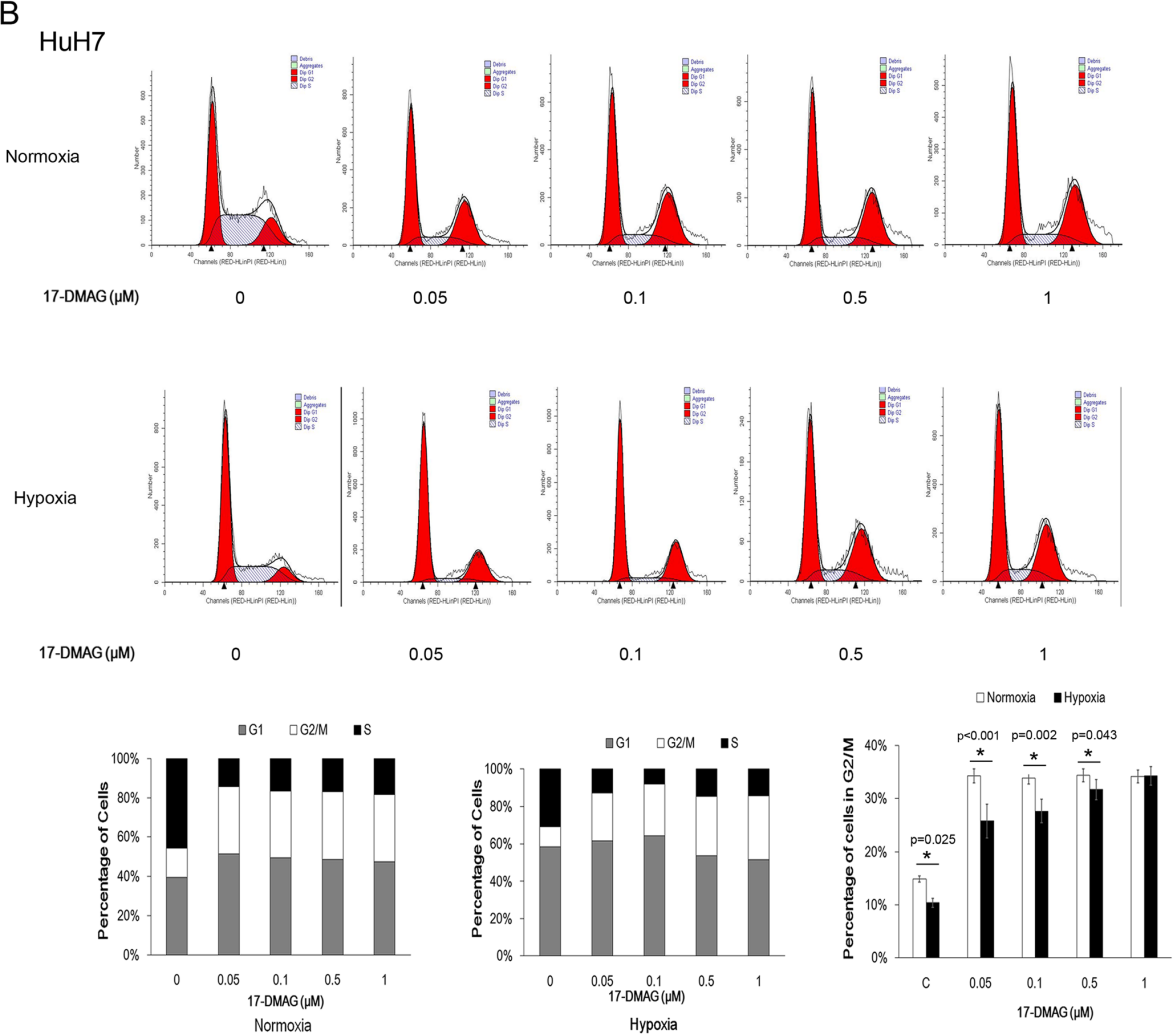
Hypoxia alleviated 17-DMAG-induced G2/M arrest. Liver cancer cell line HepG2 (**a**) and HuH7 (**b**) were treated with increasing concentrations of 17-DMAG (0, 0.05, 0.1, 0.5, 1 μM) for 24 h. Cell cycle distribution was analyzed with flow cytometry. Assays were performed in triplicate. 17-DMAG-induced G2/M arrest was more obvious in normoxia than hypoxia under 0.05, 0.1, and 0.5 μM in both cell lines, except 1 μM 17-DMAG-induced similar G2/M arrest of HuH7 cells in normoxia and hypoxia condition. Data are presented as mean ± SD of three independent experiments (*p < 0.05, hypoxia vs. normoxia)

Previous studies have demonstrated that the Hsp90 inhibitor 17-DMAG induces the G2/M blockage in certain types of human cancer cells (Watanabe et al. 2009). To investigate whether hypoxia protects the cell from 17-DMAG-induced G2/M blockages, cell cycle was examined after 24-h treatment of 17-DMAG under normoxia or hypoxia. 17-DMAG induced significant G2/M arrest under normoxia, but G2/M arrest percentage was lower in hypoxia than normoxia (Fig. 2). Under 17-DMAG concentrations of 0.1, 0.5, and 1.0 μM, HepG2 cells presented the lower G2/M arrest under hypoxia, compared with normoxia (Fig. 2a). Huh7 (Fig. 2b) presented the G2/M arrest inhibition under hypoxia, and with the increase of the concentration of 17-DMAG, from 0.05 to 0.5 μM, the difference (*P* < 0.05) of G2/M arrest between normoxia and hypoxia decreased. Only under 1 μM of 17-DMAG was the difference between normoxia and hypoxia diminished.

### 17-DMAG induced the increasing level of cyclin B1, but hypoxia attenuated the increase

**Fig. 3:**
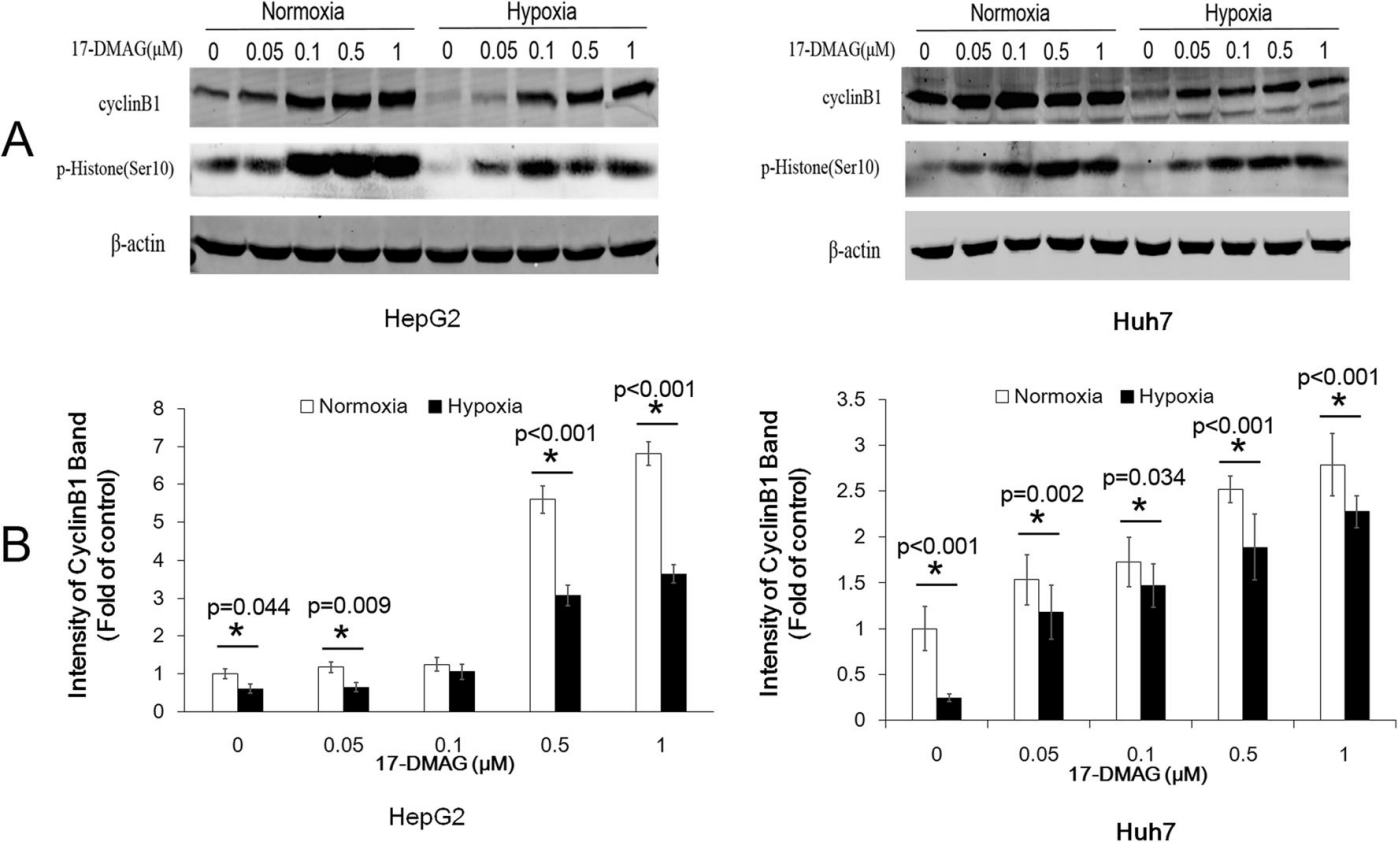
Hypoxia attenuated the 17-DMAG-induced cyclinB1 accumulation. HepG2 and Huh7 cells were incubated with 17-DMAG at different concentrations for 24 h. **a** Protein extracts were analyzed by immunoblots for cyclin B1 and p-histone 3 (Ser 10). **b** The intensities of cyclin B1 bands were analyzed by ImageJ. Data are presented as mean ± SD of three independent experiments (*p < 0.05, hypoxia vs. normoxia)

To further explore the mechanism of hypoxia-attenuated G2/M cell cycle arrest induced by 17-DMAG, we detected the cell cycle-related protein, cyclin B1, and p-histone (Ser10), which increased during G2/M phase (Fig. 3a). 17-DMAG treatment under normoxia increased the level of cyclin B1 and p-Histon3 (Ser10). Meanwhile, compared with normoxia, cyclin B1 and p-histone 3 (Ser10) induced by 17-DMAG were lower in hypoxia (Fig. 3b), which is consistent with the G2/M phase changes determined by flow cytometry.

### Hypoxia inhibited transcription of cyclin B1 and accelerated ubiquitination of cyclin B1

The dynamic change of cyclin B1 includes the delicate balance of synthesis and degradation, which is important for the cell cycle transition from G2 to M. Cyclin B1 begins to accumulate in the S phase and is localized in the cytoplasm, then reaches the maximal level at mitosis, and is imported into nucleus where it will be rapidly degraded at the meta-phase-anaphase transition (Smits and Medema 2001; Nimeus-Malmstrom et al. 2010).

**Fig. 4:**
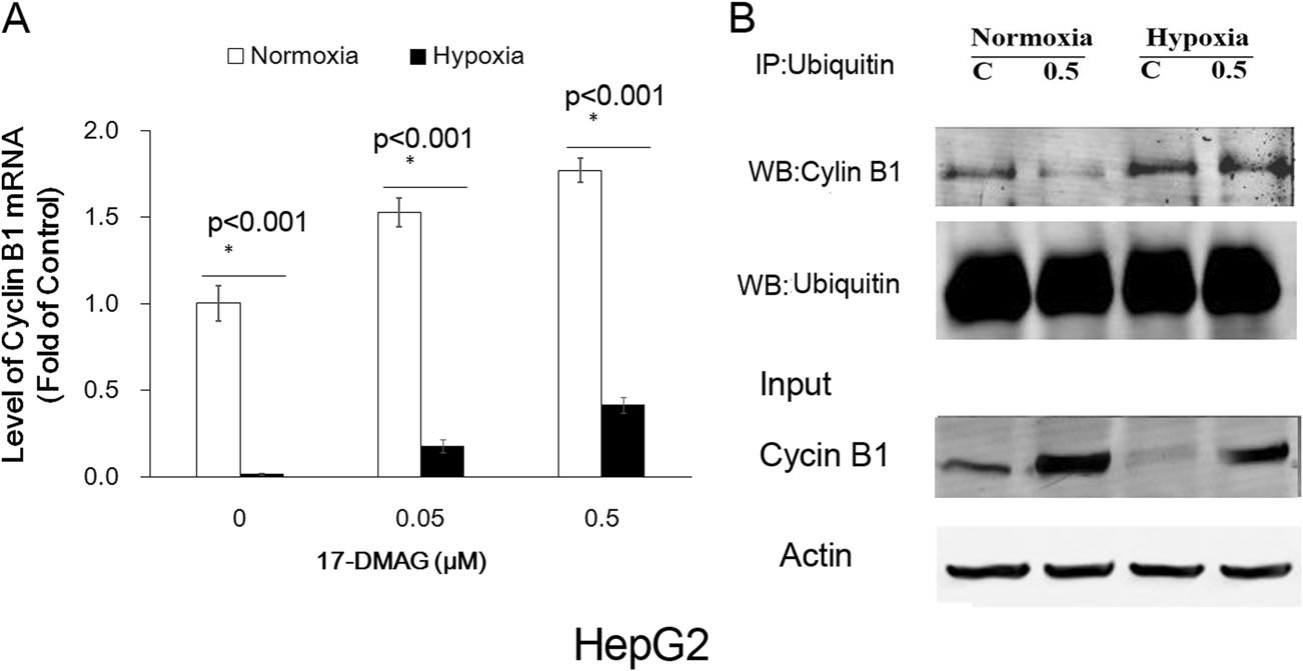
Hypoxia inhibited transcription of cyclin B1 and accelerated ubiquitination of cyclin B1. **a** The mRNA levels of cyclinB1 in HepG2 cells under normoxia or hypoxia condition for 24 h were determined by real-time PCR assay. Each experiment was done three separate times (*bars*, SD; *p < 0.05). **b** Ubiquitinated cyclin B1 was co-immunoprecipitated with mouse anti-ubiquitin antibody and probed with rabbit anti-cyclin B1 and rabbit anti-ubiquitin antibody. 17-DMAG treatment inhibited the ubiquitination of cyclin B1, and hypoxia abrogated the inhibition of cyclin B1 ubiquitination with 17-DMAG treatment

To determine why cyclin B1 decreased under hypoxia, we detected the cyclin B1 mRNA by real-time semiquantitative reverse transcriptase (RT-PCR). RT-PCR was used to determine whether decreased cyclin B1 was due to decreased transcription of cyclin B1 mRNA after 24-h exposure to hypoxia. As shown in Fig. 4a, although cyclin B1 mRNA level increased significantly when treated with 17-DMAG (0.05, 0.5 μM) under normoxia for 24 h, cyclin B1 mRNA level slightly increased under hypoxia.

Since the impairment of degradation of cyclin B1could also be contributed to cyclin B1 accumulation, the ubiquitination of cyclin B1 after 17-DMAG treatment was detected (Fig. 4b). These results indicate that 17-DMAG treatment inhibited the ubiquitination of cyclin B1 under normoxia and that hypoxia abrogated the decreased ubiquitination of cyclin B1 caused by 17-DMAG treatment.

### Hypoxia accelerated the decrease of total cyclin B1 level and the translocation of cyclin B1 into nucleus

**Fig. 5:**
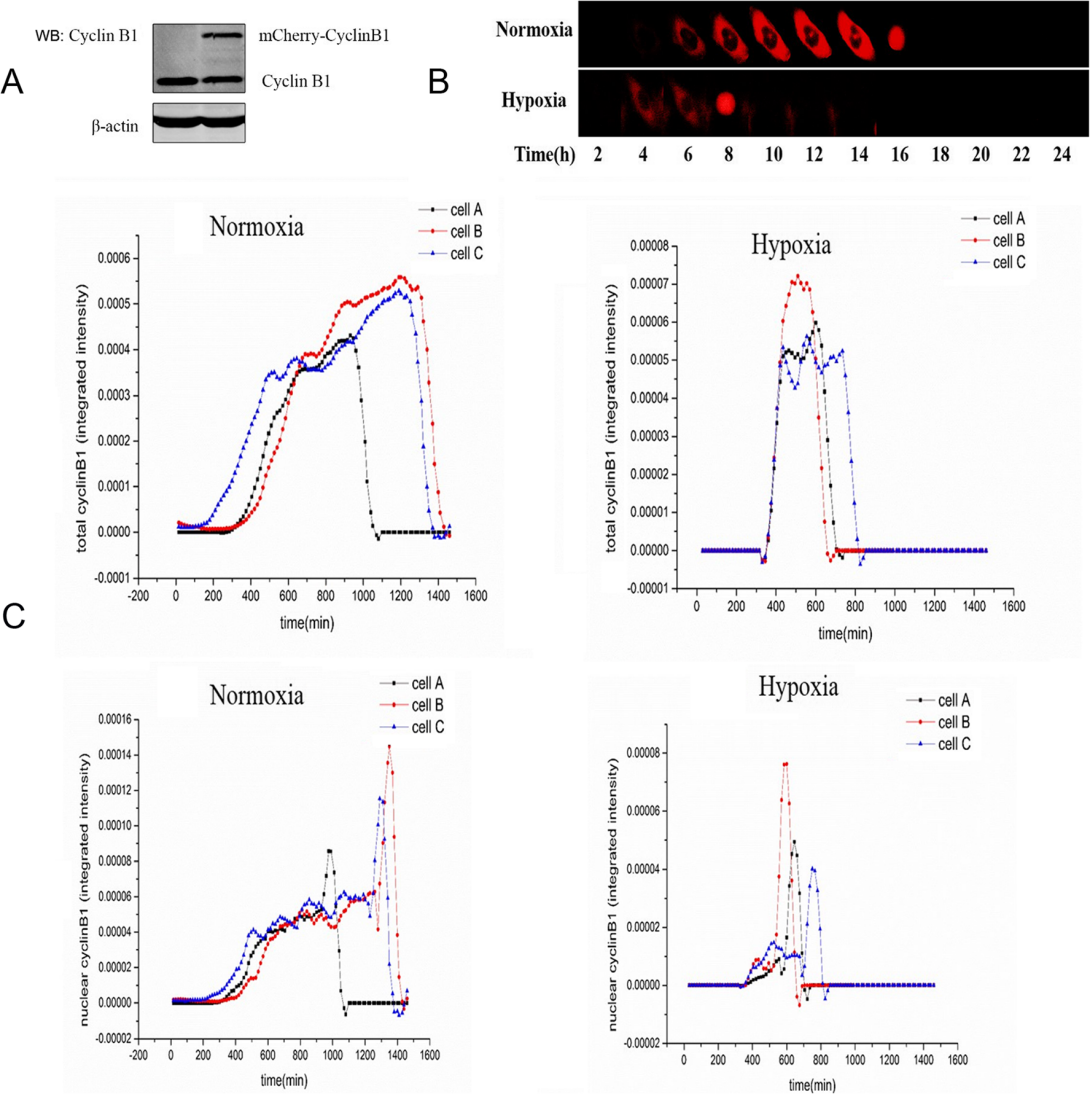
Hypoxia accelerated nuclear import and consequential degradation of cyclinB1. **a** HepG2 cells expressing cyclin B1-mCherry were checked by Western blot. **b** HepG2 cells were synchronized by serum starvation and kept in a cell chamber to mimic normoxia or hypoxia condition. The images were captured every 15 min by time-lapse florescence microscopy. One cell entering mitosis under normoxia or hypoxia treatment is displayed. The results showed that exogenous mCherry-cyclin B1 accumulated more obviously under normoxia in comparison with hypoxia. Furthermore, HepG2 cells under normoxia took longer time (16 h) to translocate and accumulate cyclin B1 into nucleus than hypoxia (8 h). Cyclin B1 rapidly degraded when cyclin B1 translocated from cytoplasm to the nucleus, which indicated the beginning of mitosis. **c** The time-lapse recording of cyclin B1 intensity was presented by three cells of each normoxia or hypoxia group for total cyclin B1 level (*upper panel*) or nuclear cyclin B1 level (**c**, *lower panel*). The experiments about the cyclin B1 nuclear translocation were repeated three times, and the similar results were observed. The three cells were from the same experiment to minimize the discrepancy caused by different experiments. The changes in the curves indicate that hypoxia accelerated time course for the decrease of total cyclin B1 level and the translocation of cyclin B1 into nucleus

To further explore the degradation and translocalization of cyclin B1 induced by hypoxia, we recorded the time-lapse images in HepG2 cells transfected with plasmid of mCherry-cyclin B1 by confocal microscopy for 24 h. The exogenous mCherry-tagged cyclin B1 was used to monitor the degradation of cyclin B1 under different treatments. Single-cell clone stably transfected with mCherry-cyclin B1 was confirmed by Western blotting (Fig. 5a). The results showed that exogenous mCherry-cyclin B1 accumulated more obviously under normoxia in comparison with hypoxia (Fig. 5b). Furthermore, HepG2 cells under normoxia took longer (16 h) to translocate and accumulate cyclin B1 into nucleus than hypoxia (8 h). Cyclin B1 rapidly degraded when cyclin B1 translocated from cytoplasm to the nucleus, which indicted the beginning of mitosis. The time-lapse recording of cyclin B1 intensity was presented by three cells of each normoxia or hypoxia group (Fig. 5c) for total cyclin B1 level (Fig. 5c, upper panel) or nuclear cyclin B1 level (Fig. 5c, lower panel). The changes in the curves indicate that hypoxia accelerated time course for the decrease of total cyclin B1 level and the translocation of cyclin B1 into nucleus.

## Discussion

Most cancer cells have deregulated G1 checkpoints making them dependent on their S and G2 checkpoints, so S and G2 checkpoints become an attractive target therapy of cancer (Chen et al. 2012). 17-AAG abrogates hepatocellular cancer growth through G2/M cell cycle arrest (Watanabe et al. 2009). 17-DMAG is a water-soluble analogue of 17-AAG (Kummar et al. 2010), and our results show that hypoxia decreases the chemosensitivity to 17-DMAG and attenuates 17-DMAG-induced G2/M arrest, with lower cyclin B1 level.

Hypoxia in tumors is associated with selection of genotypes favoring survival and pro-survival changes in gene expression and gain resistance to chemotherapy (Wilson and Hay 2011; Dong et al. 2012). Many genetic abnormalities have been revealed in tumor cells and altered expression of cell cycle regulators like cyclin D1 and cyclin-dependent kinase (CDKs). Cyclin B1 may be related to the prognosis of the patients, though the evidences are still controversial. The occurrence, development, and treatment of cancer may also be related to cyclin B1. Deregulated expression of cyclin B1 may lead to a disrupted control of cell growth and a malignant phenotype (Egloff et al. 2006; Chen et al. 2009). Abundance of cyclin B1 (Porter et al. 2000) and increased expression of cyclin B1 (Gomez et al. 2007) are parallel to better prognosis.

As a client protein of Hsp90, cyclin B1 is critical for the transition from G2 to M phase. The main function of Hsp90 is to help the folding of the denatured client proteins, and the inhibition of Hsp90 will induce the degradation of the client proteins. However, the client protein of Hsp90, cyclin B1, slightly increased in presence of Hsp90 inhibitor geldanamycin (GA) or in Hsp90 mutants, and Hsp90α is critical for the precise localization of cyclin B1 to the mitotic spindle in *Drosophila* and human cells (Basto et al. 2007). Also, treatment with Hsp90 inhibitors or heat shock induced an increasing mRNA level of cyclin B1 and the accumulation of cyclin B1 (Watanabe et al. 2009; Burrows et al. 2004). All these indicate that Hsp90 might also be involved into the cell cycle control of tumor cells and, by regulation, the level of cyclin B1. And, then what is the relevance between chemoresistance and cyclin B1? In our research, the results suggest that 17-DMAG induces accumulation of cyclin B1 and inhibits tumor cell proliferation. Meanwhile, hypoxia impairs the 17-DMAG-induced proliferation inhibition with lower cyclin B1 level through inhibiting cyclin B1 mRNA transcription and accelerating cyclin B1 nuclear translocation and degradation. Since cyclin B1 nuclear translocation and degradation are critical for G2/M transition and cell division, decreased cyclin B1 level could be tightly related to hypoxia-induced chemoresistance to 17-DMAG.

Our research provides a possible mechanism of hypoxia-induced chemoresistance in liver tumor, and the decrease level of cyclin B1 coordinates with the chemoresistance to Hsp90 inhibitor 17-DMAG under hypoxia. Since the presence of a quinone of 17-DMAG makes it uniquely redox sensitive (Samuni et al. 2010). How much does it contribute to hypoxia-induced chemoresistance in liver tumor? How does hypoxia inhibit cyclin B1 level? Is it caused by hypoxia-induced DNA damage and genotype adaptation? All these still need to be further explored with other Hsp90 inhibitors in different liver tumor cell lines and in vivo experiments.

## References

[CR1] Barrott JJ, Haystead TA (2013). Hsp90, an unlikely ally in the war on cancer. FEBS J.

[CR2] Basto R, Gergely F, Draviam VM, Ohkura H, Liley K, Raff JW (2007). Hsp90 is required to localise cyclin B and Msps/ch-TOG to the mitotic spindle in Drosophila and humans. J Cell Sci.

[CR3] Black JC, Atabakhsh E, Kim J, Biette KM, Van Rechem C, Ladd B, Burrowes PD, Donado C, Mattoo H, Kleinstiver BP, Song B, Andriani G, Joung JK, Iliopoulos O, Montagna C, Pillai S, Getz G, Whetstine JR (2015). Hypoxia drives transient site-specific copy gain and drug-resistant gene expression. Genes Dev.

[CR4] Burrows F, Zhang H, Kamal A (2004). Hsp90 activation and cell cycle regulation. Cell Cycle.

[CR5] Chen X, Kang H, Zou F (2009). Low concentration of GA activates a preconditioning response in HepG2 cells during oxidative stress-roles of Hsp90 and vimentin. Cell Stress Chaperones.

[CR6] Chen T, Stephens PA, Middleton FK, Curtin NJ (2012). Targeting the S and G2 checkpoint to treat cancer. Drug Discov Today.

[CR7] Dong XL, Xu PF, Miao C, Fu ZY, Li QP, Tang PY, Wang T (2012). Hypoxia decreased chemosensitivity of breast cancer cell line MCF-7 to paclitaxel through cyclin B1. Biomed Pharmacother.

[CR8] Egloff AM, Vella LA, Finn OJ (2006). Cyclin B1 and other cyclins as tumor antigens in immunosurveillance and immunotherapy of cancer. Cancer Res.

[CR9] Gavet O, Pines J (2010). Activation of cyclin B1-Cdk1 synchronizes events in the nucleus and the cytoplasm at mitosis. J Cell Biol.

[CR10] Ghattass K, Assah R, El-Sabban M, Gali-Muhtasib H (2013). Targeting hypoxia for sensitization of tumors to radio- and chemotherapy. Curr Cancer Drug Targets.

[CR11] Gomez LA, de Las PA, Reiner T, Burnstein K, Perez-Stable C (2007). Increased expression of cyclin B1 sensitizes prostate cancer cells to apoptosis induced by chemotherapy. Mol Cancer Ther.

[CR12] Gwak GY, Yoon JH, Kim KM, Lee HS, Chung JW, Gores GJ (2005). Hypoxia stimulates proliferation of human hepatoma cells through the induction of hexokinase II expression. J Hepatol.

[CR13] Harrison L, Blackwell K (2004). Hypoxia and anemia: factors in decreased sensitivity to radiation therapy and chemotherapy?. Oncologist.

[CR14] Horsman MR, Mortensen LS, Petersen JB, Busk M, Overgaard J (2012). Imaging hypoxia to improve radiotherapy outcome. Nat Rev Clin Oncol.

[CR15] Isaacs JS, Xu W, Neckers L (2003). Heat shock protein 90 as a molecular target for cancer therapeutics. Cancer Cell.

[CR16] Karakashev SV, Reginato MJ (2015). Progress toward overcoming hypoxia-induced resistance to solid tumor therapy. Cancer Manag Res.

[CR17] Kummar S, Gutierrez ME, Gardner ER, Chen X, Figg WD, Zajac-Kaye M, Chen M, Steinberg SM, Muir CA, Yancey MA, Horneffer YR, Juwara L, Melillo G, Ivy SP, Merino M, Neckers L, Steeg PS, Conley BA, Giaccone G, Doroshow JH, Murgo AJ (2010). Phase I trial of 17-dimethylaminoethylamino-17-demethoxygeldanamycin (17-DMAG), a heat shock protein inhibitor, administered twice weekly in patients with advanced malignancies. Eur J Cancer.

[CR18] Lin S, Hoffmann K, Schemmer P (2012). Treatment of hepatocellular carcinoma: a systematic review. Liver Cancer.

[CR19] Nimeus-Malmstrom E, Koliadi A, Ahlin C, Holmqvist M, Holmberg L, Amini RM, Jirstrom K, Warnberg F, Blomqvist C, Ferno M, Fjallskog ML (2010). Cyclin B1 is a prognostic proliferation marker with a high reproducibility in a population-based lymph node negative breast cancer cohort. Int J Cancer.

[CR20] Olcina M, Lecane PS, Hammond EM (2010). Targeting hypoxic cells through the DNA damage response. Clin Cancer Res.

[CR21] Ortiz B, Porras F, Jimenez-Martinez MC, Montano LF, Martinez-Cairo S, Lascurain R, Zenteno E (2002). Differential expression of a 70 kDa O-glycoprotein on T cells: a possible marker for naive and early activated murine T cells. Cell Immunol.

[CR22] Pacey S, Wilson RH, Walton M, Eatock MM, Hardcastle A, Zetterlund A, Arkenau HT, Moreno-Farre J, Banerji U, Roels B, Peachey H, Aherne W, de Bono JS, Raynaud F, Workman P, Judson I (2011). A phase I study of the heat shock protein 90 inhibitor alvespimycin (17-DMAG) given intravenously to patients with advanced solid tumors. Clin Cancer Res.

[CR23] Patki JM, Pawar SS (2013). HSP90: chaperone-me-not. Pathol Oncol Res.

[CR24] Porter LA, Singh G, Lee JM (2000). Abundance of cyclin B1 regulates gamma-radiation-induced apoptosis. Blood.

[CR25] Samuni Y, Ishii H, Hyodo F, Samuni U, Krishna MC, Goldstein S, Mitchell JB (2010). Reactive oxygen species mediate hepatotoxicity induced by the Hsp90 inhibitor geldanamycin and its analogs. Free Radic Biol Med.

[CR26] Smits VA, Medema RH (2001). Checking out the G(2)/M transition. Biochim Biophys Acta.

[CR27] Venook AP, Papandreou C, Furuse J, de Guevara LL (2010). The incidence and epidemiology of hepatocellular carcinoma: a global and regional perspective. Oncologist.

[CR28] Watanabe G, Behrns KE, Kim JS, Kim RD (2009). Heat shock protein 90 inhibition abrogates hepatocellular cancer growth through cdc2-mediated G2/M cell cycle arrest and apoptosis. Cancer Chemother Pharmacol.

[CR29] Wilson WR, Hay MP (2011). Targeting hypoxia in cancer therapy. Nat Rev Cancer.

[CR30] Yan W, Fu Y, Tian D, Liao J, Liu M, Wang B, Xia L, Zhu Q, Luo M (2009). PI3 kinase/Akt signaling mediates epithelial-mesenchymal transition in hypoxic hepatocellular carcinoma cells. Biochem Biophys Res Commun.

[CR31] Yan W, Chang Y, Liang X, Cardinal JS, Huang H, Thorne SH, Monga SP, Geller DA, Lotze MT, Tsung A (2012). High-mobility group box 1 activates caspase-1 and promotes hepatocellular carcinoma invasiveness and metastases. Hepatology.

[CR32] Zeng H, Zheng R, Guo Y, Zhang S, Zou X, Wang N, Zhang L, Tang J, Chen J, Wei K, Huang S, Wang J, Yu L, Zhao D, Song G, Chen J, Shen Y, Yang X, Gu X, Jin F, Li Q, Li Y, Ge H, Zhu F, Dong J, Guo G, Wu M, Du L, Sun X, He Y, Coleman MP, Baade P, Chen W, Yu XQ (2015). Cancer survival in China, 2003–2005: a population-based study. Int J Cancer.

